# Systematic Review with Meta-Analysis: Fecal Calprotectin as a Surrogate Marker for Predicting Relapse in Adults with Ulcerative Colitis

**DOI:** 10.1155/2019/2136501

**Published:** 2019-05-28

**Authors:** Jiajia Li, Xiaojing Zhao, Xueting Li, Meijiao Lu, Hongjie Zhang

**Affiliations:** Department of Gastroenterology, The First Affiliated Hospital of Nanjing Medical University, Nanjing, Jiangsu Province 210029, China

## Abstract

The clinical course of ulcerative colitis (UC) is featured by remission and relapse, which remains unpredictable. Recent studies revealed that fecal calprotectin (FC) could predict clinical relapse for UC patients in remission, which has not yet been well accepted. To detect the predictive value of FC for clinical relapse in adult UC patients based on updated literature, we carried out a comprehensive electronic search of PubMed, Web of Science, Embase, and the Cochrane Library to identify all eligible studies. Diagnostic accuracy including pooled sensitivity, specificity, positive likelihood ratio (PLR), negative likelihood ratio (NLR), diagnostic odds ratio (DOR), and pooled area under the receiver operating characteristic (AUROC) was calculated using a random effects model. Heterogeneity across studies was assessed by the *I*^2^ metric. Sources of heterogeneity were detected using subgroup analysis. Metaregression was used to test potential factors correlated to DOR. Publication bias was assessed using Deek's funnel plots. In our study, 14 articles enrolling a total of 1110 participants were finally included, and all articles underwent a quality assessment. Pooled sensitivity, specificity, PLR, and NLR with 95% confidence intervals (CIs) were 0.75 (95% CI: 0.70–0.79), 0.77 (95% CI: 0.74–0.80), 3.45 (95% CI: 2.31–5.14), and 0.37 (95% CI: 0.28–0.49) respectively. The area under the summary receiver operating characteristic (sROC) curve was 0.82, and the diagnostic odds ratio was 10.54 (95% CI: 6.16–18.02). Our study suggested that FC is useful in predicting clinical relapse for adult UC patients in remission as a simple and noninvasive marker.

## 1. Introduction

Ulcerative colitis (UC), one subtype of inflammatory bowel disease (IBD), which is characterized by chronic mucosal inflammation, affects more than 1 million people in the United States and Europe [1, 2]. The etiology of UC still remains incompletely clear. Studies suggest that intestinal microbial dysbiosis, host genetics, and external environment may play an important role in triggering UC's chronic inflammation and in determining its subsequent disease behavior and outcomes [3–5]. The clinical course of UC is featured by remission and relapse, which remains unpredictable [6]. Uncertain clinical recurrence will affect the life quality of UC patients and require extended therapy as well as extra medical costs [7]. If we were able to identify patients with a high risk of clinical flare-up, adjusted treatment at a presymptomatic stage could be carried out. Therefore, an earlier prediction of possible relapse is urgently needed for clinical physicians. Generally, endoscopy together with histological examination is considered the standard for assessing UC relapse [8]. However, as an invasive method, it is often intolerable and inconvenient, which limits its use in predicting UC relapse [9]. A simple, reliable, and readily available test is needed to detect an imminent flare for timely escalation of treatment and better disease control.

Fecal calprotectin (FC), a calcium-combined protein, mainly derives from neutrophil cells during inflammation. The concentration of FC reflects the extent of neutrophil migration to the gastrointestinal tract [10]. It is becoming the most useful noninvasive tool for monitoring the inflammatory status of the mucosa and for assessing patients' response to therapy [11–13]. However, the role of FC as a predictor of clinical relapse in UC patients remains controversial. In the present study, we aim to pool the updated literature in this field and try to figure out the predictive value of FC for clinical relapse in adult UC patients.

## 2. Materials and Methods

This meta-analysis was conducted in accordance with the Preferred Reporting Items for Systematic Reviews and Meta-Analyses (PRISMA) statement [14].

### 2.1. Literature Search

Databases including PubMed, Web of Science, Embase, and the Cochrane Library were searched up to December 31, 2018 to identify all eligible studies. To avoid omission of potentially useful articles, we used both Medical Subject Heading (MeSH) terms and free words, including “Inflammatory Bowel Disease;” “Bowel Diseases, Inflammatory;” “Colitis, Ulcerative;” “ulcerative colitis;” “UC;” “calprotectin;” “Leukocyte L1 complex;” “relapse;” “recrudescence;” “recur;” “recrudesce;” and “recurrence.” Meanwhile, previous systematic reviews and meta-analysis were also explored to seek for potential relevant studies. No language restriction was involved in the search strategy.

### 2.2. Study Selection

A study was included if it met the following criteria: [1] prospective studies used FC to predict UC relapse, [2] FC level for predicting UC relapse was measured at remission, [3] estimates of diagnostic accuracy (such as sensitivity or specificity) were provided, [4] the identification of relapse was based on clinical symptoms or endoscopic findings, and [5] studies were conducted in adult populations. Two reviewers (Jiajia Li and Xiaojing Zhao) independently reviewed the search results to determine article inclusion while screening the citations. In cases of discordance, a consensus was reached through discussion with another author (Xueting Li). Studies were excluded if they were in consonance with any of the following: [1] patients were diagnosed with other coexisting gastroenterological diseases and [2] studies not separating UC from other IBDs like Crohn's Disease (CD) and Inflammatory Bowel Disease Unclassified (IBD-U).

### 2.3. Data Extraction

To ensure accuracy, the quantitative data were collected independently by two investigators. All forms of data were extracted using a standard form, including general information (name of the first author, year of publication, and population characteristics), FC assay, test results, cutoff value, and follow-up time. Test results were presented as the numbers of true positive (TP), false positive (FP), false negative (FN), and true negative (TN) for each study.

### 2.4. Quality Assessment

Two authors rated each selected study for quality according to the QUADAS-2 (Quality Assessment of Diagnostic Accuracy Studies) tool, which is recommended by the Cochrane Diagnostic Reviewers' Handbook [15]. The QUADAS-2 checklist comprises 4 parts of quality assessment: patient selection, index test, reference standard, and flow and timing. For the first three parts, they each contain two aspects: risk of bias and concerns regarding applicability, while the last part only contains risk of bias. Disagreement was resolved by a consensus.

### 2.5. Statistical Analysis

Statistical analyses were performed with Meta-DiSc statistical software v. 1.4 (Universidad Complutense, Madrid, Spain) and Stata statistical software v. 12.0 (StataCorp, College Station, TX). First, for each study, sensitivity, specificity, positive likelihood ratio (PLR), negative likelihood ratio (NLR), and diagnostic odds ratio (DOR) were calculated after constructing a diagnostic 2 × 2 table. Then, pooled estimates of all included studies with 95% confidence intervals (CIs) were calculated using a DerSimonian-Laird random-effect model. For threshold analysis, correlation between sensitivity and specificity (presented as logit true positive rate (TPR) vs. logit false positive rate (FPR)) was tested to explore threshold effects, and the Moses-Shapiro-Littenberg mode was used to assess constant DOR. After that, a summary receiver operating characteristic (sROC) curve was performed, and depending on whether the DOR is constant, a symmetrical or asymmetrical sROC was used [16]. Heterogeneity across studies was assessed by the *I*^2^ metric. Statistically, *I*^2^ > 50% indicates that the heterogeneity is significant and the random effects model should be used. Otherwise, the fixed-effect model should be adopted [17, 18]. To investigate the source of heterogeneity, subgroup analysis was conducted. Preplanned subgroups were defined according to the number of patients in the respective studies (<80 or ≥80), mean age (<40 or ≥40), male ratio (<50% or ≥50%), FC assay (Bühlmann or non-Bühlmann), FC cutoff (<150 *μ*g/g or ≥150 *μ*g/g), and follow-up time (<1 y or ≥1 y). Based on that, potential factors correlated to DOR were also tested by metaregression. Finally, publication bias was tested using Deek's funnel plot [19]. Continuous values were presented as mean ± standard deviation or a range and discrete variables as numbers and percentages. *P* value<0.05 was considered statistically significant.

## 3. Results

### 3.1. Study Selection and Characteristics of Included Studies

**Figure 1:**
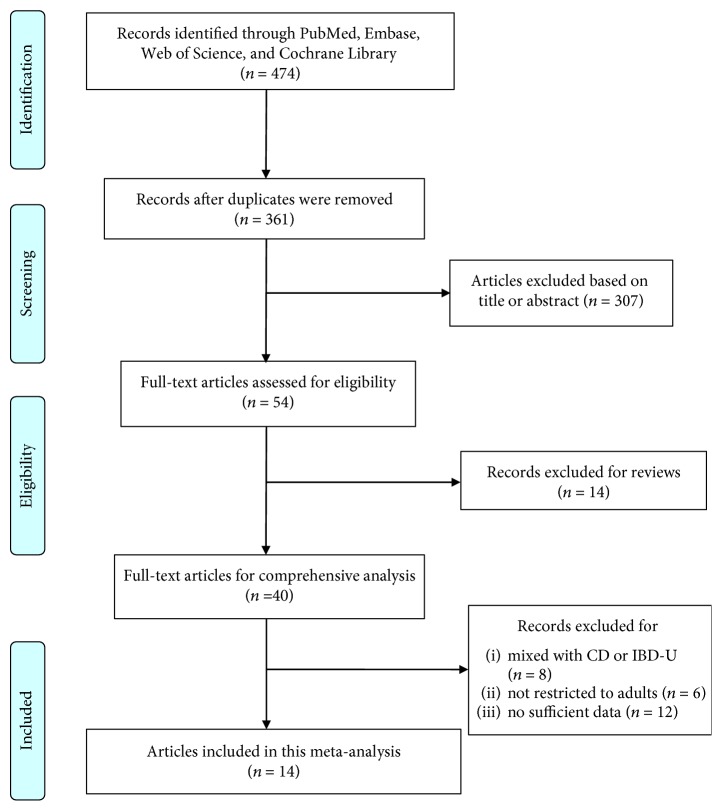
Study flow diagram showing the process of selecting studies concerning the diagnostic accuracy of FC in predicting relapse among adult UC patients.

The flow diagram of the study selection is summarized in Figure 1. The search strategy yielded 474 articles. After the removal of 113 duplicates, 361 citations remained. Then, based on the title or abstract, 307 citations were excluded and the remaining 54 articles were further scanned. 14 articles were excluded for reviews. Another 26 studies were removed for not being restricted to adults, mixing with diseases like CD and IBD-U, or not providing sufficient data. Finally, 14 articles enrolling a total of 1110 subjects were eligible for our meta-analysis [20–33]. The baseline characteristics of the included studies are shown in Table 1.

### 3.2. Assessment of Methodological Quality of the Included Studies

**Figure 2:**
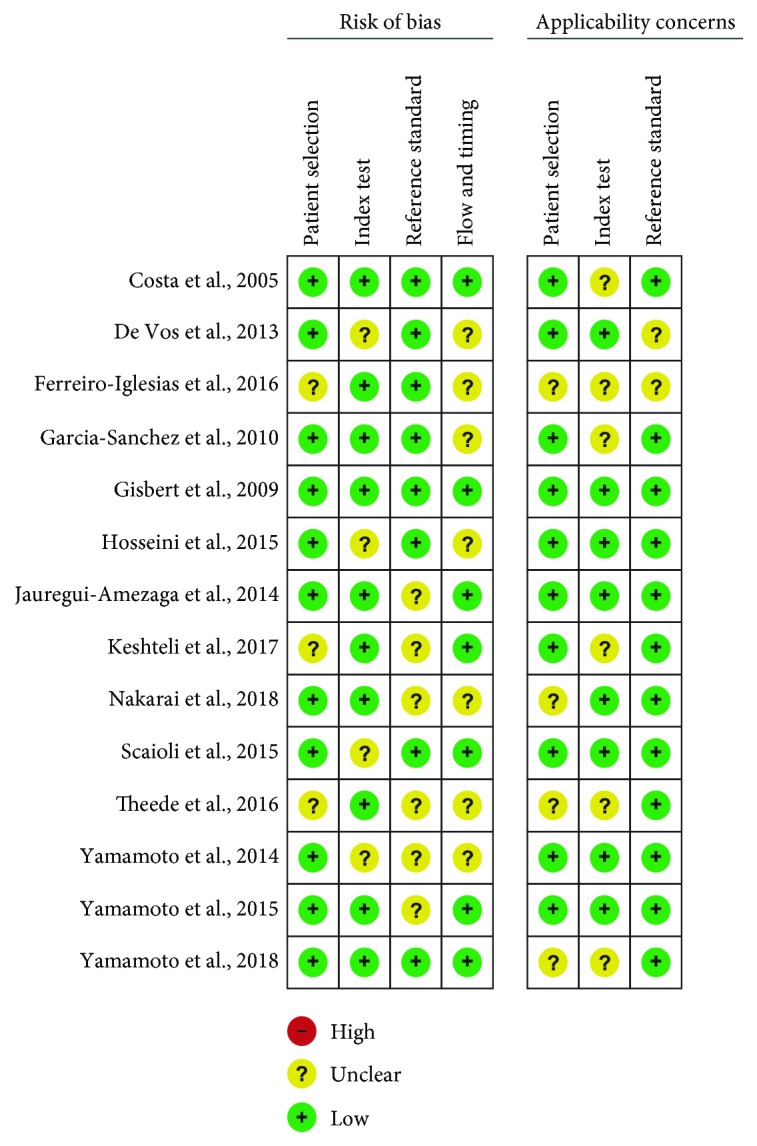
QUADAS-2 risk of bias assessment. +, high; −, low; ?, unclear.

The 14 studies underwent quality assessment using the QUADAS-2 tool. All trials included in our study yielded good quality, thus the pooled results should be persuasive. A summary of the results is presented in Figure 2.

### 3.3. Diagnostic Accuracy Meta-Analysis

**Figure 3:**
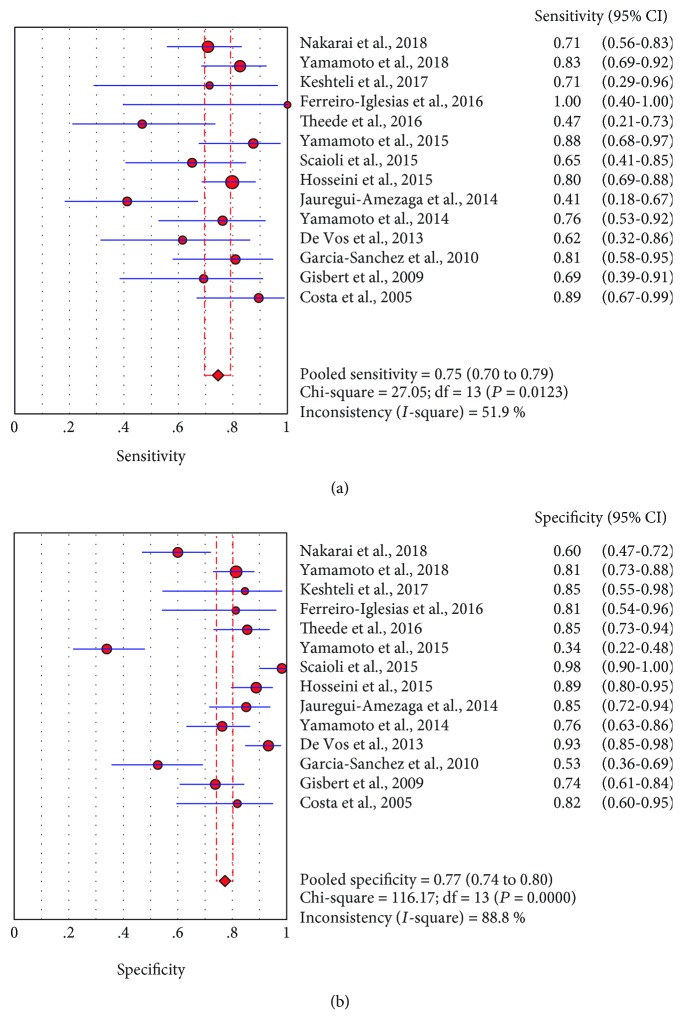
Forest plots of pooled sensitivity of FC in predicting relapse of UC in one-year follow-up (a). Forest plots of pooled specificity of FC in predicting relapse of UC (b).

Forest plots in Figures 3(a) and 3(b) show the pooled sensitivity and specificity. The sensitivity ranged from 0.41 to 1 (pooled sensitivity 0.75, 95% CI: 0.70–0.79), while specificity ranged from 0.34 to 0.98 (pooled specificity 0.77, 95% CI: 0.74–0.80). The pooled PLR was 3.45 (95% CI: 2.31–5.14), NLR was 0.37 (95% CI: 0.28–0.49), and DOR was 10.54 (95% CI: 6.16–18.02) (Table 2).

**Figure 4:**
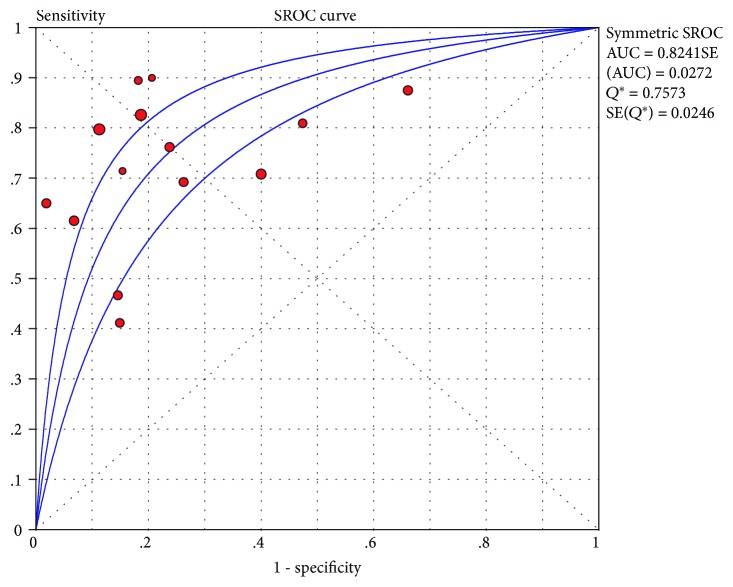
Symmetrical summary receiver operator curve (sROC) for all 14 studies. The size of the circle represents the sample size of each study included in the meta-analysis.

The Spearman correlation coefficient for logit (TPR) vs. logit (FPR) was 0.503 (*P* = 0.067), indicating that the correlation between the TPR and FPR was not significant. The Moses-Shapiro-Littenberg method showed that DOR was constant (*b* = −0.239, *P* = 0.212). Therefore, a symmetrical sROC was appropriate to calculate the diagnostic accuracy (Figure 4). As shown in Figure 4, the area under the receiver operator curve (AUC) (SE) is 0.82 (0.027) and the *Q* statistic (SE) is 0.76 (0.025).

### 3.4. Subgroup Analysis and Metaregression

For all 14 studies, the heterogeneity (*I*^2^) was 51.9% (sensitivity), 88.8% (specificity), 85.4% (positive LR), 51.6% (negative LR), and 57.0% (DOR). To detect possible factors contributing to heterogeneity, subgroup analysis was performed (Table 2). Factors including the number of patients, age, male/female ratio, FC assay, cutoff value, and follow-up time are all possible sources of heterogeneity. The heterogeneity of DOR was lower in studies with a male/female ratio < 50% (*I*^2^, 0% vs. 67.7%) and studies with a mean age < 40 (*I*^2^, 34.9% vs. 62.3%). Studies with a smaller sample size showed lower heterogeneity (*I*^2^, 39.5% vs. 72.5%). FC was more diagnostically accurate in studies using Bühlmann as the FC assay (DOR = 15.35; 95% CI: 5.28-44.61) compared with studies using other assays (DOR = 9.34; 95% CI: 5.06-17.24). In addition, FC showed more diagnostic accuracy in studies with longer follow-up time (DOR = 11.18; 95% CI: 6.32-19.78) and a larger cutoff value (DOR = 14.06; 95% CI: 7.17-27.58).

To see if there are any covariates correlated to DOR, we performed metaregression. The factors included the following domains: the number of patients, age, male/female ratio, FC assay, cutoff value, and follow-up time. No significant correlation between the covariates and DOR was detected in the univariate metaregression analysis (Table 3).

### 3.5. Publication Bias Analysis

**Figure 5:**
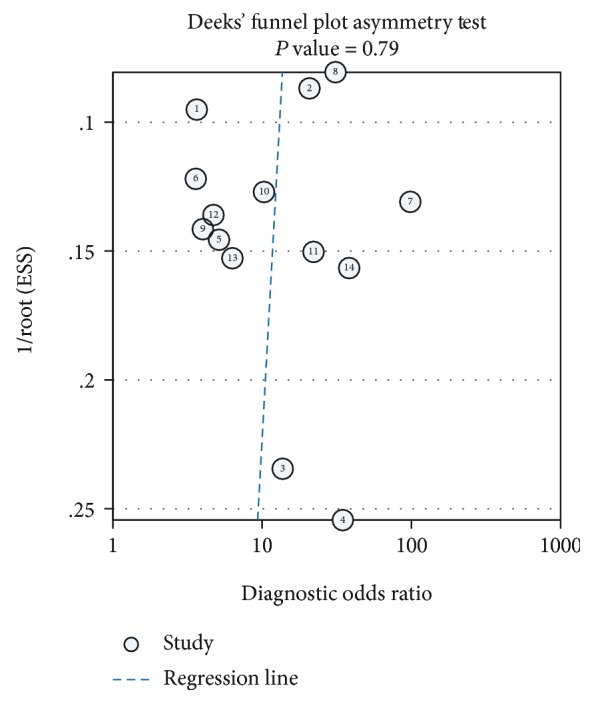
Deeks' funnel plot.

Deek's funnel plot asymmetry test was used to assess the publication bias of the included studies (Figure 5), and no obvious publication bias was detected (*P* = 0.79).

## 4. Discussion

The clinical course of UC is characterized by periods of remission with recurrent episodes of symptom exacerbation because of acute intestinal inflammation [34]. Presently, UC remains an incurable disease, and the aim of existing treatments is to induce remission, promote the healing of the mucosal membrane, and decrease the incident of relapse [35]. Relapses in UC are hard to predict, and the identification of patients with a high risk of clinical flare-up could lead to target treatment at a presymptomatic stage. To better monitor the course of UC, FC has been proposed as a reliable biomarker for the prediction of possible relapse in patients with remission [36].

Our study revealed that FC yielded a good prediction value for the clinical relapse of UC (with a pooled sensitivity and specificity of 0.75, 95% CI: 0.70–0.79 and 0.77, 95% CI: 0.74–0.80, respectively). The maximum joint sensitivity and specificity was 0.76 (SE 0.025), with an AUC of 0.82 (SE 0.027). These findings are consistent with a previous meta-analysis [37].

No consensus has been reached for the definition of the clinical relapse of UC, and the criteria adopted in our studies were not identical. The basic criterion of clinical relapse is worsening of symptoms. Apart from that, other indices include TW score ≥ 11 [32, 33] and partial Mayo score ≥ 3 [20, 22, 23]; the others focused on the Mayo endoscopic subscore. This could be a source of heterogeneity. We noticed that the specificity in the study of Scaioli et al. [26] is extremely high (100%). This may result from the relatively loose relapsing standard set in the study. To date, the golden standard for assessing intestinal inflammation is histological examination [38]. A recent study conducted by Diamanti et al. validated that a FC value of 275 *μ*g/g achieved sensitivity and negative predictive value of 97% and specificity and positive predictive value of 85% in predicting the histological relapse of IBD [39]. Though using a histological standard could more accurately demonstrate the diagnostic value of FC, the clinical value of this study was limited, because histological relapse does not necessarily develop to clinical relapse and may not need adjustment of medication. Therefore, a more accurate, well-recognized standard of clinical relapse should be established.

The FC value adopted in our analysis was the baseline value tested at enrollment. However, elevated FC is not synonymous to bowel inflammation and FC levels are subject to some day-to-day and diurnal variations. Thus, many studies held that consecutive fecal calprotectin measurements could better predict relapse in patients with UC [40]. De Vos et al. suggested that two consecutive measurements >300 mg/kg were more specific than a single measurement for predicting relapse [30]. Consecutive fecal calprotectin measurements can monitor the disease status. The possibility of clinical relapse is increased if the value of fecal calprotectin stays abnormal. So if patients can keep a regular outpatient visit during remission, we suggest that they take consecutive fecal calprotectin measurements.

Subgroup analysis shows that the diagnostic accuracy is higher in studies with longer follow-up time (≥1 year), compared with those with shorter follow-up time. This suggests that FC is more useful in predicting long-term outcome. Since the disease course of UC is chronic, this finding will help monitor UC patients in the long run.

Patients included in this analysis are restricted to adults. However, in clinical practice, pediatric UC patients make up an important part and are drawing daily increasing attention [41]. Relevant studies show that fecal calprotectin can also serve as an activity marker of IBD in children [42, 43]. Walkiewicz et al. found that among children with CD in remission, FC levels may be useful in predicting impending clinical relapse. In their study, eighty-nine percent of CD encounters with FC levels less than 400 *μ*g/g remained in clinical remission [44]. However, it has been reported that the reference ranges of FC in children are age-related and vary a lot [45]. Besides, the clinical characteristics differ a lot between pediatric and adult UC patients. Therefore, only articles restricted to adult patients were included in our meta-analysis. To demonstrate FC's role in predicting relapse in pediatric UC patients, more clinical studies should be conducted.

Recently, the quantitative fecal immunochemical test (FIT) is proposed as a surrogate method to predict relapse in ulcerative colitis [46, 47]. FIT holds several advantages over FC in regard to user friendliness, including a lower cost, easy and clean handling, and the ability to make rapid measurements by using an automated measurement system [48]. Moreover, studies confirmed that if FIT was applied together with FC and other biomarkers like CRP, the diagnostic accuracy would be significantly improved [49, 50]. However, the present data remains insufficient and further studies regarding the combination of FIT and FC for predicting relapse of UC are warranted.

## 5. Conclusion

Our results confirm the diagnostic utility of FC for the detection of UC relapse in adults. Due to its simplicity and noninvasiveness, measuring FC levels at clinical remission appears to be a reliable and reproducible indicator for predicting UC relapse. To further explore its utility, more well-designed studies are required to confirm our results and find the best cutoff value of FC concentration to identify recurrence in UC patients with remission.

## Figures and Tables

**Table 1:** Characteristics of the included studies.

Study	Year	Number of patients	Age (year)	Male (%)	FC assay	Standard of relapse	Follow-up time	Results				
								TP	FP	FN	TN	Cutoff (*μ*g/g)
Nakarai et al. [20]	2018	113	16–82	50%	PhiCal	WS, partial Mayo score ≥ 3	2 y	34	26	14	39	75
Yamamoto et al. [21]	2018	164	31-39	61.59%	Cell Sciences	WS, MES ≥ 2	1 y	38	22	8	96	115
Keshteli et al. [22]	2017	20	42.7 ± 18.8	45%	Bühlmann	WS, partial Mayo score ≥ 3	1 y	5	2	2	11	124
Ferreiro-Iglesias et al. [23]	2016	20	19-68	40%	Bühlmann	WS, partial Mayo score ≥ 3	8 w	4	3	0	13	198
Theede et al. [24]	2016	70	39.3 ± 13.9	72.90%	Bühlmann	WS	1 y	7	8	8	47	321
Yamamoto et al. [27]	2015	80	18–74	58.80%	Cell Sciences	WS, MES ≥ 1	8 w	21	37	3	19	55
Scaioli et al. [26]	2015	74	43.2 ± 17.9	72%	Calprest	WS, Mayo score > 3	1 y	13	1	7	53	193
Hosseini et al. [25]	2015	154	42 ± 10	51.30%	Bühlmann	WS, Seo index > 220	1 y	59	9	15	71	341
Jauregui-Amezaga et al. [28]	2014	64	46 ± 15.3	79%	Cerba Internacional	WS, MES ≥ 1	1 y	7	7	10	40	250
Yamamoto et al. [29]	2014	80	35.1 ± 0.8	61%	Cell Sciences	WS, MES ≥ 2	1 y	16	14	5	45	170
De Vos et al. [30]	2013	87	48 ± 15	45%	PhiCal	WS, MES ≥ 2	1 y	8	5	5	69	300
García-Sánchez et al. [33]	2010	69	40.4 ± 13.1	59%	Calprest	WS, TW score ≥ 11	1 y	17	18	4	20	120
Gisbert et al. [32]	2009	74	43 ± 13	48%	PhiCal	WS, TW score ≥ 11	1 y	9	16	4	45	164
Costa et al. [31]	2005	41	24-54	71%	Calprest	WS, Mayo score > 3	1 y	17	4	2	18	150

TP: true positive; FP: false positive; FN: false negative; TN: true negative; WS: worsening of symptoms; TW: modified Truelove-Witts score; ET, Edwards and Truelove score; MES: Mayo Endoscopic Subscore. PhiCal, Bühlmann, Cell Sciences, Calprest, and Cerba Internacional are different fecal calprotectin test kits.

**Table 2:** Assessment of diagnostic accuracy and heterogeneity in subgroup analysis.

Category	Number of studies	Sensitivity	Specificity	PLR	NLR	DOR	*I* ^2^ (%) of DOR
Total	14	0.75 (0.70–0.79)	0.77 (0.74–0.80)	3.45 (2.31–5.14)	0.37 (0.28–0.49)	10.54 (6.16–18.02)	57.0
Number of patients							
≥80	6	0.78 (0.72-0.83)	0.75 (0.71-0.79)	3.39 (1.77-6.51)	0.32 (0.23-0.43)	11.18 (4.96-25.19)	72.5
<80	8	0.68 (0.59-0.76)	0.81 (0.76-0.85)	3.38 (2.10-5.43)	0.44 (0.30-0.63)	9.60 (4.57-20.15)	39.5
Mean age							
≥40	10	0.73 (0.67-0.79)	0.75 (0.71-0.79)	3.34 (2.00-5.58)	0.40 (0.30-0.53)	9.61 (4.75-19.47)	62.3
<40	4	0.77 (0.68-0.85)	0.81 (0.76-0.86)	3.92 (2.96-5.20)	0.30 (0.15-0.62)	13.44 (6.31-28.60)	34.9
Male ratio							
≥50%	10	0.75 (0.70-0.80)	0.75 (0.72-0.79)	3.71 (1.97-5.10)	0.37 (0.26-0.52)	9.98 (5.16-19.32)	67.7
<50%	4	0.70 (0.53-0.84)	0.84 (0.78-0.89)	4.31 (2.38-7.80)	0.39 (0.24-0.62)	12.49 (5.31-29.37)	0
FC assay							
Bühlmann	4	0.75 (0.65-0.83)	0.87 (0.80-0.91)	5.07 (3.32-7.75)	0.34 (0.16-0.72)	15.35 (5.28-44.61)	44.8
Not Bühlmann	10	0.74 (0.68-0.80)	0.75 (0.71-0.78)	3.05 (1.98-4.69)	0.38 (0.28-0.52)	9.34 (5.06-17.24)	58.0
Cutoff value							
≥150 *μ*g/g	9	0.71 (0.65-0.78)	0.86 (0.82-0.89)	4.42 (3.00-6.53)	0.38 (0.26-0.55)	14.06 (7.17-27.58)	49.9
<150 *μ*g/g	5	0.79 (0.71-0.85)	0.64 (0.58-0.69)	2.19 (1.32-3.64)	0.36 (0.25-0.50)	6.72 (2.95-15.47)	59.8
Follow-up time							
≥1 y	12	0.73 (0.68-0.78)	0.81 (0.78-0.84)	3.66 (2.52-5.31)	0.38 (0.28-0.50)	11.18 (6.32-19.78)	59.8
<1 y	2	0.89 (0.72-0.98)	0.44 (0.33-0.57)	2.20 (0.66-7.28)	0.31 (0.11-0.88)	7.01 (0.92-53.25)	41.0

**Table 3:** Results of univariate metaregression analysis of diagnostic odds ratio.

Covariables	*P* value	RDOR	95% CI
Number of patients (≥80/<80)	0.65	1.41	(0.24-8.31)
Mean age (≥40/<40)	0.68	0.69	(0.09-5.50)
Male/female ratio (≥50%/<50%)	0.74	0.73	(0.08-6.77)
FC assay (Bühlmann/not Bühlmann)	0.80	1.25	(0.16-9.60)
Cutoff value (≥150 *μ*g/g/<150 *μ*g/g)	0.68	1.50	(0.15-15.17)
Follow-up time (≥1 y/<1 y)	0.92	0.85	(0.02-30.36)

RDOR, relative DOR.
